# Altered Resting State in Diabetic Neuropathic Pain

**DOI:** 10.1371/journal.pone.0004542

**Published:** 2009-02-20

**Authors:** Franco Cauda, Katiuscia Sacco, Sergio Duca, Dario Cocito, Federico D'Agata, Giuliano C. Geminiani, Sergio Canavero

**Affiliations:** 1 CCS fMRI, Koelliker Hospital, Turin, Italy; 2 Department of Psychology, University of Turin, Turin, Italy; 3 Department of Neuroscience, AOU San Giovanni Battista, Turin, Italy; 4 Turin Advanced Neuromodulation Group, Turin, Italy; Tel Aviv University, Israel

## Abstract

**Background:**

The spontaneous component of neuropathic pain (NP) has not been explored sufficiently with neuroimaging techniques, given the difficulty to coax out the brain components that sustain background ongoing pain. Here, we address for the first time the correlates of this component in an fMRI study of a group of eight patients suffering from diabetic neuropathic pain and eight healthy control subjects. Specifically, we studied the functional connectivity that is associated with spontaneous neuropathic pain with spatial independent component analysis (sICA).

**Principal Findings:**

Functional connectivity analyses revealed a cortical network consisting of two anti-correlated patterns: one includes the left fusiform gyrus, the left lingual gyrus, the left inferior temporal gyrus, the right inferior occipital gyrus, the dorsal anterior cingulate cortex bilaterally, the pre and postcentral gyrus bilaterally, in which its activity is correlated negatively with pain and positively with the controls; the other includes the left precuneus, dorsolateral prefrontal, frontopolar cortex (both bilaterally), right superior frontal gyrus, left inferior frontal gyrus, thalami, both insulae, inferior parietal lobuli, right mammillary body, and a small area in the left brainstem, in which its activity is correlated positively with pain and negatively with the controls. Furthermore, a power spectra analyses revealed group differences in the frequency bands wherein the sICA signal was decomposed: patients' spectra are shifted towards higher frequencies.

**Conclusion:**

In conclusion, we have characterized here for the first time a functional network of brain areas that mark the spontaneous component of NP. Pain is the result of aberrant default mode functional connectivity.

## Introduction

Living with chronic pain is highly maladaptive: several studies have shown that chronic pain can modify the way in which one perceives their everyday experience, in turn generating negative emotions and thoughts and changing the physiological and psychological processes [1]–[3]. The underlying mechanism between pain and cognitive function is not completely understood but it likely involves some common cortical elements that are related to attentional, self monitoring, and emotional networks.

Over the past 15 years, the brain correlates of neuropathic pain (NP) have been characterized in several neuroimaging studies [4]. In fact, these studies focused on the evoked components of NP (e.g. allodynia), given the relative simplicity of setting up an ad hoc protocol, namely, subtracting the activated state resulting from the painful stimulus from the basal reference state. However, evoked pain is not the defining trait of NP: spontaneous, background pain is. Only a few SPECT and PET studies have investigated this component, as a standard functional MRI (fMRI) is unable to explore the background activity of the brain [5], [6]. The drawback of all these techniques is that they do not explore the global interconnectedness of the brain areas that are involved in pain processing.

Few studies have explored the effect of pain on brain functional connectivity [7]–[11], in which there is little known as to how pain modulates brain networks, which operate together at rest as a functional unit [7], [9].

Several cortical areas have been implicated in pain processing but, puzzlingly enough, both activations and deactivations have been observed, the significance of which in the genesis of background NP has not been coherently explained [5], [12]. Up to now, correlating these findings with the mechanism of spontaneous pain has remained, to a large extent, unfruitful. Some studies have suggested that low-frequency fluctuations in resting fMRI data that were collected using blood oxygen level dependent (BOLD) contrast in turn correspond to functionally relevant resting state networks (RSN), which are a major source of non-modelled signals in BOLD fMRI data [13]–[15]. It has been shown that a brain at rest is characterized by an organized baseline or default mode network (DMN), which is suspended during specific goal-oriented (task-positive) mental activity [16]. This DMN has been implicated in an introspectively oriented self-referential mode of mental activity, e.g. self-awareness, sensory input processing, and self-monitoring [14] and has been suggested as a potential marker in the diagnosis of several neurological and psychiatric disorders [17]–[20].

Pain perception emerges from the flow and integration of information between specific brain areas and, therefore, lends itself to connectivity analyses. The underlying assumption of the present study is that, under conditions that generate background NP, the resting, “default” state is altered. Thus, we compared the DMN of a sample of diabetic neuropathic pain patients with that of a group of matched normal controls adopting a spatial Independent Component Analysis (sICA) approach [21]–[23]. While conducting our experiment, another study based on a similar assumption was published [7], showing that chronic back pain is associated with disturbed DMN dynamics. However, some important differences exist between that and our work: Baliki and collaborators' studied functional connectivity indirectly, extracting it from an activation task (i.e. attention fMRI paradigm); their patients suffered form back pain; their results were obtained from deactivations and seed voxel correlations. Moreover, in our study, as brain activity engages intercommunicating networks [24] with different patterns of synchrony [25]–[27] along different oscillatory bands, and brain disorders disrupt such synchronization patterns [28], we also explored DMN in terms of frequency bands.

## Materials and Methods

### Ethics Statement

All of the subjects gave their informed written consent, in line with the Declaration of Helsinki, and the study was approved by the local ethics committee.

### Patients

Eight right-handed consecutive patients suffering from peripheral NP (diabetic pain) (four women and four men; age range = 51–78, mean age = 61 years) were enrolled from our multidisciplinary pain unit (Table 1).

**Table 1:** Demographics of patients

Patient	Pain duration	Paresthesias	Dysesthesias	Quality of pain	VAS (0–10)	Distribution
1	2 yrs	+		Pulsating-burning	6	Lower limbs
2	2 yrs	+	+	Burning -	5	Lower limbs
3	3 yrs		+	Burning-squeezing	5	Lower limbs
4	3 yrs	+	+	Burning-squeezing	6	Lower limbs
5	2 yrs	+		Lancinating-burning	6	Lower limbs
6	2 yrs	+		Burning-squeezing	4	Lower limbs
7	3 yrs		+	Burning-piercing	9	Lower limbs
8	5 yrs	+		Burning-piercing	4	Lower limbs

All patients underwent a complete neurological and psychological examination as well as standard MR brain scanning by an experienced Neuroradiologist (SD) to exclude structural/white-matter abnormalities on anatomical MR-images. Patients suffering from significant psychiatric disorders were excluded. All of the patients were assessed using standardized pain scales (Visual Analog Scale - VAS, Numerical Rating Scale - NRS, McGill Pain Questionnaire - Italian version). The spontaneous component of their pain syndrome was evaluated on the MPQ checklist. VAS readings were obtained from their clinical records both the day before and on the day of the study. In all cases, pain was restricted to the lower limbs. The duration of pain was >2 years in all the cases. Patients were washed out of their medications at least one month before imaging (opioids or cannabinoids were never administered). At the time of the scanning, the pain intensity had reached the pre-treatment levels. Maximum care was taken to avoid situations that could actually trigger evoked pain during the imaging sessions. Eight age- and gender-matched right-handed healthy volunteers (four women and four men; age range = 47–79, mean age = 59 years) acted as controls. None suffered from any neurological or psychiatric disorders, including chronic pain of any kind, and they also did not have a history of drug or alcohol abuse. None were on medications that are known to alter brain activity. All of the females participating in the study were menopausal.

### Task and image acquisition

All of the subjects were instructed simply to keep their eyes closed, think of nothing in particular, and not to fall asleep.

Data acquisition was performed on a 1.5 Tesla INTERA™ scanner (Philips Medical Systems) with a SENSE high-field, high resolution (MRIDC) head coil that was optimized for functional imaging. Resting state functional T2*-weighted images were acquired using echoplanar (EPI) sequences, with a repetition time (TR) of 2000 ms, an echo time (TE) of 50 ms, and a 90° flip angle. The acquisition matrix was 64×64, and the field of view (FoV) 200 mm. A total of 200 volumes were acquired; each volume consisted of 19 axial slices, parallel to the anterior-posterior (AC-PC) commissure line and covering the whole brain; slice thickness was 4.5 mm with a 0.5 mm gap. Two scans were added at the beginning of the functional scanning and the data was discarded to reach a steady-state magnetization before acquiring the experimental data.

In the same session, a set of three-dimensional high-resolution T_1_-weighted structural images was acquired for each participant. This data-set was acquired using a Fast Field Echo (FFE) sequence, with a repetition time (TR) of 25 ms, ultra-short echo time (TE), and a 30° flip angle. The acquisition matrix was 256×256, and the field of view (FoV) 256 mm. The set consisted of 160 contiguous sagittal images covering the whole brain. In-plane resolution was 1×1 mm and slice thickness 1 mm (1×1×1 mm voxels).

### Data analysis

BOLD imaging data were analyzed using the Brain Voyager QX software (Brain Innovation, Maastricht, Holland); a plug-in extension of this software was used to compute the individual functional connectivity analyses. The functional data of each subject underwent the following pre-processing steps: 1) Mean intensity adjustment to prevent global signal variability; 2) slice scan time correction, using a sinc interpolation algorithm; 3) 3D motion correction: all of the volumes were aligned spatially to the first volume by rigid body transformations, using a trilinear interpolation algorithm; 4) spatial smoothing using a Gaussian kernel of 4 mm FWHM; 5) temporal filters (i.e. linear trend removal and non-linear trend removal using a temporal high-pass filter [frequency pass = 0.008 Hz]) were applied to remove drifts due to scanner, and other, low frequency noises.

After pre-processing, a series of steps were followed in order to allow for the precise anatomical location of brain activity to facilitate inter-subject averaging. First, each subject's slice-based functional scan was co-registered on his or her 3D high-resolution structural scan. This process involved mathematical co-registration exploiting the slice positioning stored in the headers of the raw data, as well as fine adjustments that were computed by comparing the data sets based on their intensity values; if needed, manual adjustments were also performed. Second, the 3D structural data set of each subject was transformed into Talairach space: the cerebrum was translated and rotated into the anterior-posterior commissure plane and then the borders of the cerebrum were identified. Third, using the anatomo-functional coregistration matrix and the determined Talairach reference points, the functional time course of each subject was transformed into Talairach space and the volume time course was created. The Talairach transformation was performed in two steps. The first step consisted of rotating the 3D data set of each subject to align it with the stereotactic axes. In the second step, the extreme points of the cerebrum were specified. These points were then used to scale the 3D data sets to the dimensions of the standard brain of the Talairach and Tournoux atlas using a piecewise affine and continuous transformation for each of the 12 defined subvolumes. The individual maps were projected onto the average volumetric image to be displayed using volumetric anatomy.

Functional connectivity was measured using independent component analysis (ICA), which is a statistical technique that separates a set of signals into independent uncorrelated and non-Gaussian spatio-temporal components [22]. The fMRI brain image at each time point was treated as a mixture of spatial independent components; sICA extracts the different components, each with its unique time course, maximizing their spatial statistical independence. In order to limit the sICA decomposition to the voxels lying within the parenchyma, a mask based on an average image from the functional data sets of all the subjects was created: For each subject, only those voxels that were within such a mask were included in the analysis.

The ICA decomposition was calculated using the single-subject ICA plug-in, which corresponded to a C++ implementation of the fast-ICA algorithm [29]. The correct number of components to be extracted was estimated using the Rissanen minimum description length or MDL information-theoretic criteria [30]. The initial dimensions of the functional data set were reduced from 200 (number of time points) to the number previously calculated with MDL (mean number of components 38; min 28; max 41) using the principal component analysis (PCA) technique, which accounted in all the subjects for more than 99% of the total variance-covariance. Then, for each subject, the independent components were estimated using the fast-ICA method, which minimizes the mutual information of the components using a robust approximation of the neg-entropy as a contrast function and a fast, iterative algorithm for its maximization [31], [29]. The resulting components were thresholded (z>2.5, cluster size = ten or more contiguous voxels).

### Selection of the components

After the ICA was performed, three criteria were used to select the component that most closely matched the default-mode network. 1) ICA components were first screened for the presence of frequencies lower than 0.008Hz and higher than 0.1 Hz by rejecting those components having more than 25% of their total power spectrum outside the frequency range of 0.008–0.1 Hz . 2) ICA components were then screened for the presence of the remaining motion or global signal variability by calculating the regression between those nuisance factors and each IC time course; the components with a correlation of r>0.20 with the nuisance factors were discarded. 3) A spatial template of the default mode network, based on previous related studies, e.g. [32], was used to select the best fit of the remaining components. In particular, in using the ICA plug-in, we spatially correlated all the components with a default mode mask that was generated by WFU Pick-atlas [33]. This mask contained the posterior parietal cortex (Brodmann's area 7), frontal pole (Brodmann's area 10), and occipitoparietal junction (Brodmann's area 39), as well as the posterior cingulate and precuneus. It was smoothed with a Gaussian kernel of 4 mm FWHM. The component that spatially correlated most significantly with the template was selected as the default mode component.

### Group statistical map

The group components were calculated as random effects maps. The random effects statistic for each voxel of the z-maps was generated by the ICA plugin and was calculated as the mean z-value of that voxel across the individual maps divided by its standard error, in turn resulting in a t-statistic; the resulting map of the t-values was visualized by using a (p<0.05) one-sided FDR corrected threshold. A two-sample t-test was used to compare the healthy subjects' and the patients' group maps.

Significant clusters of activation for the two sample t tests were determined by using a (p<0.05) FDR corrected threshold.

## Results

**Figure 1:**
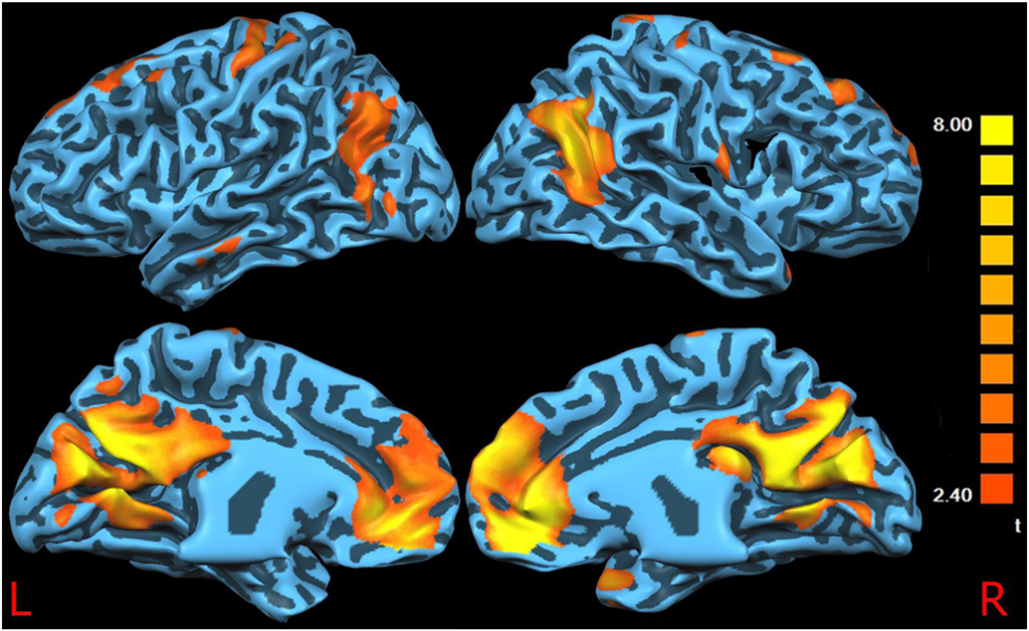
Controls DMN. Surface-rendered projection of the Default Mode Network components found in the control subjects.

**Figure 2:**
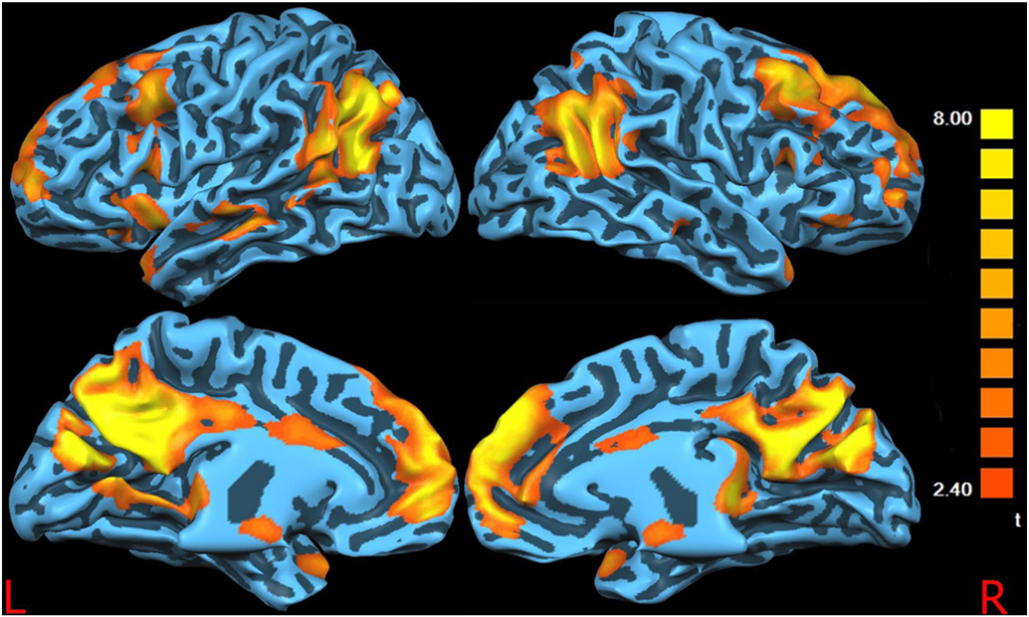
Patients DMN. Surface-rendered projection of the Default Mode Network components found in the patients.

Figures 1 and 2 show the surface-rendered projection of the RSN components found in the control subjects and patients, respectively. There is a significant overlap between the two images and the areas previously described in the literature as a part of the resting functional connectivity across the majority of clusters including the posterior cingulate cortex, anterior cingulate cortex, bilateral inferior parietal cortex, medial prefrontal cortex, and dorsolateral prefrontal cortex. As it has been suggested that age and gender can affect the resting state activation patterns [34], [35], we compared males vs. females and older (age>55) vs. younger (age<55) subjects' connectivity maps. No statistically significant differences emerged from these comparisons (data not shown).

**Figure 3:**
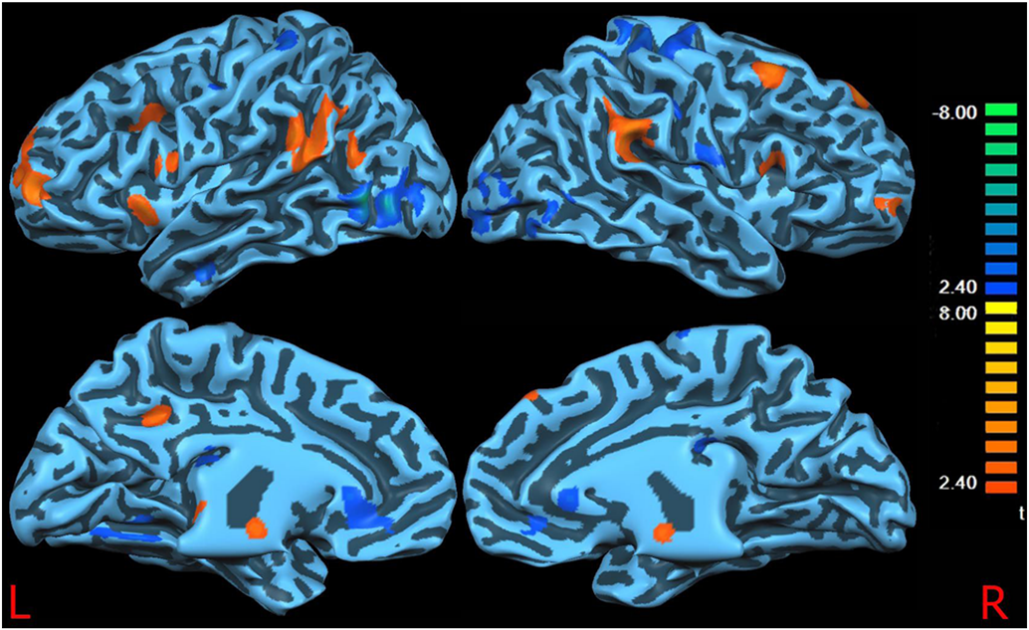
DMN differences between controls and patients. Surface-rendered projection results of a two-sample t-test contrasting the Default Mode Network in the healthy group vs. the pain group. The blue foci indicate the areas that showed significantly less correlational activity in the pain group than in the healthy group. Vice versa the yellow/red foci indicate the areas that showed significantly more correlational activity in the pain group than in the healthy group.

Functional connectivity between-groups comparison (Figure 3; Tables 2–3)

**Table 2:** Brain areas of increased connectivity in pain subjects vs. healthy subjects

x	y	z	Brodmann's area	Location	Cluster size	Hemisphere
49	16	19	9	Inferior Frontal Gyrus	865	R
−53	19	15	45	Inferior Frontal Gyrus	1429	L
30	54	4	10	Middle Frontal Gyrus	1356	R
−22	57	8	10	Middle Frontal Gyrus	5012	L
−29	31	22	9	Middle Frontal Gyrus	1311	L
33	16	41	8	Right Middle Frontal Gyrus	2041	R
10	42	42	8	Right Superior Frontal Gyrus	1139	R
33	21	9	13	Insula	418	R
−35	17	2	13	Insula	1532	L
10	−29	5		Thalamus	322	R
−8	−27	3		Thalamus	319	L
56	−41	29	40	Inferior Parietal Lobule	3857	R
−54	−42	25	40	Inferior Parietal Lobule	6198	L
−11	−43	37	31	Precuneus	964	L
4	−10	−6		Mammillary body	858	R
−4	−33	−38		Brainstem	889	L

**Table 3:** Brain areas of decreased connectivity in pain patients vs. healthy subjects

x	y	z	Brodmann's area	Location	Cluster size	Hemisphere
34	−25	58	4	Precentral Gyrus	5334	R
36	−33	60	3	Postcentral Gyrus	4911	R
−51	−11	44	3	Postcentral Gyrus	398	L
50	−3	22	6	Precentral Gyrus	10832	R
9	−12	68	6	Superior Frontal Gyrus	1117	R/L
−31	−36	63	2	Postcentral Gyrus	1014	L
−52	−12	46	3	Postcentral Gyrus	399	L
1	32	3	32	Anterior Cingulate	3632	R/L
39	−77	−5	19	Inferior Occipital Gyrus	8695	R
−44	−73	1	19	Inferior Temporal Gyrus	5642	L
−18	−64	−5	19	Lingual Gyrus	3101	L
−45	−10	−22	20	Fusiform Gyrus	596	L
−39	−61	−13	37	Fusiform Gyrus	673	L

Patients displayed a reduced connectivity in the left fusiform gyrus, left lingual gyrus, left inferior temporal gyrus, right inferior occipital gyrus, dorsal anterior cingulate cortex (dACC) bilaterally, but also the pre and postcentral gyri (SI/MI) bilaterally. Vice versa, patients displayed a greater connectivity between the left precuneus, dorsolateral prefrontal (DLPF) and frontopolar cortex (both bilaterally), right superior and left inferior frontal gyri, both thalami, both insulae, inferior parietal lobuli, right mammillary body, and a small area in the left brainstem.

### Power spectra

In order to explore the possible differences in the signal time course power density [36], we performed a power spectrum analysis on the time courses of the Independent Components (IC) that were identified as the default mode network for each subject; all of the IC time courses were sampled with the same number of data points (200). For every band in which the signal was decomposed (0.008–0.02 Hz, 0.02–0.05 Hz, 0.05–0.1 Hz, 0.1–0.25 Hz), a repeated measure ANOVA was performed with bands as a within-subjects factor (band 1–4) and groups as a between-subjects factor (patients vs. controls).

**Figure 4:**
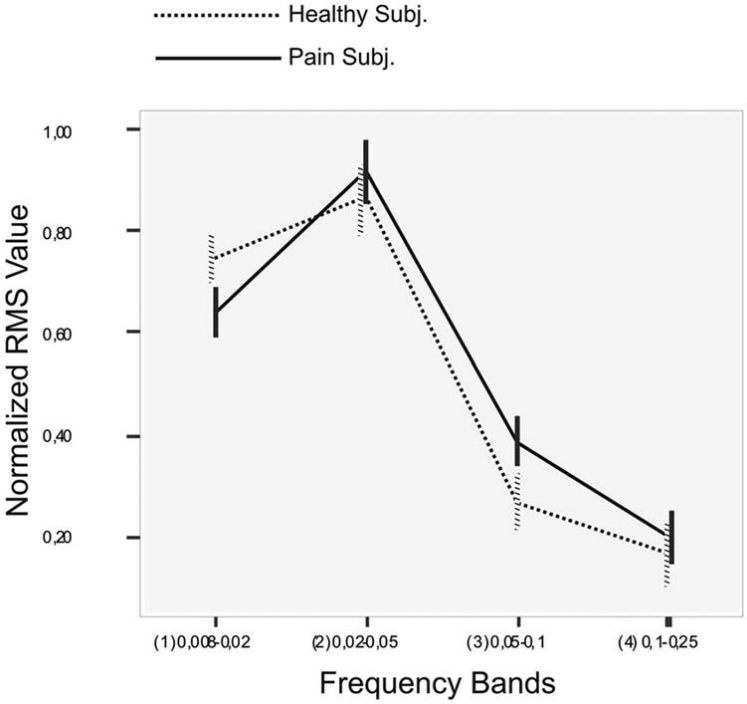
Power spectrum. Power spectrum graph of the distribution of the IC power density plot averaged across all healthy group and pain group subjects.

Power spectral analysis [37] showed a statistically significant difference between the frequency bands (F = 209.079; P<0.0001) and in the interaction between the bands and groups (F = 4.312; P = 0.01). The power spectrum graph (Figure 4) shows the distribution of the IC power density plot averaged across both the healthy group and pain group with respective standard errors to provide an indication on group variability.

A one-way ANOVA for each single frequency band shows a significant difference between the patients and controls in band 1 (0.008–0.02 Hz; F = 4.834; P = 0.03) and in band 3 (0.05–0.1 Hz; F = 5.578; P = 0.02).

In sum, the power levels in the four frequency bands demonstrated higher frequency oscillations in the pain group's IC: among the patients, the intensity curve is lower at lower frequencies and higher at higher frequencies. Although in both groups' power cluster in the 0.02–0.05 Hz range, the patients' spectra are centered at a higher frequency than those of controls.

## Discussion

The main result of the present work is that the default mode maps of those patients suffering from neuropathic pain differ significantly from those of the healthy controls. Moreover, the power spectra are shifted towards higher frequencies.

### The spontaneous component as a disrupted resting state

Within the NP group, a reduced DMN connectivity and a greater interconnection between some pain-related areas emerged. Specifically, the all-important sensori-motor areas (SI-MI) showed reduced connectivity bilaterally, as expected, while several frontal areas, insulae, and thalami showed greater connectivity, which is a sign of the postulated stronger cognitive-emotional components [5]. A chronically taxed pain-focused attentional network might play a role [9]. A functional connectivity analysis of the PET data suggests that chronic visceral pain involves areas that are related to pain affect, such as the anterior cingulate cortex and the insula [38]. A connectivity study of capsaicin-induced heat allodynia found that the left DLPF cortex is negatively correlated with the pain affect, dampening the effective connectivity in the midbrain-medial thalamic pathway; the right DLPF cortex is associated with a weakened relationship of the anterior insula with both pain intensity and effect [39]. Frontal areas are relevant in mediating or controlling the functional interactions between the key pain processing brain regions that subsequently produce changes in the perceptual correlates of pain, independent of changes in the peripheral inputs [40]. The prefrontal areas are functionally connected with the parietal regions within and between the hemispheres during the resting state [41]. This correlation appears to be strengthened in NP - as shown by our data. It seems that the altered configuration of network connectivity as postulated by recent fractal theories [42], [43] is indeed mirrored in a change of the DMN state [44], [45]. Although we cannot provide mechanistic explanations, this can support the idea of a plastic reorganization of the brain in patients that continue to suffer from chronic pain. This finding can also be related to the observation of a brain atrophy in chronic back pain [1].

Studies show that chronic NP entails a reconfiguration of network connectivity, with fragmentation of cortical processing into “islands” of decorrelated activity [46]. Decorrelation implies that neuron clusters tend to discharge relatively independently, with a partial breakdown of the estimated capability of these circuits. Each neuronal cluster involved in the nociceptive sensory pathway turns into a disorderly source of CNS input. The system is thus less flexible in sampling sensory input, i.e. in occupying different discharge frequency bands. A fixed attractor state will bias the system towards rigidity. This dissolution takes place in the lateral and medial pain streams. It is our contention that the “signature” of these restructured dynamics is mirrored in a change in the default state, as reported here and in Baliki and collaborators' study [7], who reported less deactivation in mPFC, amygdala, and PCC in pain patients vs. controls. Indeed, although the two works are not directly comparable due to the different techniques and patients involved, both support the hypotheses that chronic pain affects the overall brain function, causing an alteration of the DMN. This hypothesis finds further support in the results of a recent paper demonstrating that acupuncture modulates the resting state connectivity in the default mode network [8].

### Higher RSN frequency as marker of NP

In the present study, RSN in the pain group displayed higher frequency compared to healthy subjects. Functional connectivity studies, such as the present one, only assess a very-low frequency domain, in turn making a comparison with related studies problematic.

Oscillations between 3–10 Hz are observed mostly in SI and more variably in other cortical areas after intra-arterial, pain-inducing injection of bradykinin [47]. In particular, the sensation of acute pain is accompanied by a low-frequency (6–14 Hz) oscillation, with strong synchrony between the medial frontal cortex and SI [48]. The slow rhythm is produced by a disruption of corticothalamic feedback [5], when the EEG power is shifted towards low frequencies: In NP states, areas of theta activity abut areas of beta activity, resulting from complex patterns of hyper-hypoactivity at cortical columnar levels [49]. What we observed in our study was that in the very-low frequency domain (<1 Hz), chronic neuropathic pain tends to occupy the highest range, and the reverse of that is seen at higher frequencies. This marks a stronger synchrony between the frontal and more posterior areas (insulae, inferior parietal lobuli), which emerges when the DMN is studied.

### Weaknesses of the study

Our study addressed functional connectivity. Functional connectivity refers to the arbitrary relationships that might exist between the co-varying activity of distinct and often well-separated neuronal populations, without any reference to the physical connections or an underlying causal model, during a specific condition. A common criticism of neuroimaging is that the information obtained is correlative: Studies rarely describe causation, in which symptoms are merely being related to changes in brain activity within specialized regions. By contrast, effective connectivity refers to causal effects that one neuronal population exerts on another, and is based on an underlying model of the way the different neuronal populations are physically connected [50]–[52]. This weakness does not detract from the “core” observation that the DMN of the NP brain is “readjusted” towards a qualitatively different resting state.

Secondly, our analysis was mainly cortical. Of course, several subcortical areas play a role in the genesis of chronic NP [53] and future studies should attempt to merge cortical analysis with subcortical contributions.

Finally, peripheral NP has similar features independent of the causative factor (whether this is diabetes, herpes, or trauma). In this regard, we do not expect there to be major differences with other peripheral NP states. It will be of much interest to compare patients with central pain and PNP in future studies, as evidence suggests a difference may exist [5].

### Conclusion

These findings suggest that the brain of chronic pain patients differs from that of healthy subjects by showing a reduced default mode network and an increased resting functional connectivity in some pain related areas. This can speculatively suggest a “signature”, or marker of the spontaneous component of NP.
